# Sirtuin 2 (SIRT2): Confusing Roles in the Pathophysiology of Neurological Disorders

**DOI:** 10.3389/fnins.2021.614107

**Published:** 2021-05-24

**Authors:** Xiuqi Chen, Wenmei Lu, Danhong Wu

**Affiliations:** Department of Neurology, Shanghai Fifth People’s Hospital, Fudan University, Shanghai, China

**Keywords:** SIRT2, neuroinflammation, oxidative stress, synaptic change, axonal degeneration, autophagy, apoptosis, programmed necrosis

## Abstract

As a type of nicotinamide adenine dinucleotide (NAD^+^)-dependent deacetylases, sirtuin 2 (SIRT2) is predominantly found in the cytoplasm of cells in the central nervous system (CNS), suggesting its potential role in neurological disorders. Though SIRT2 is generally acknowledged to accelerate the development of neurological pathologies, it protects the brain from deterioration in certain circumstances. This review summarized the complex roles SIRT2 plays in the pathophysiology of diverse neurological disorders, compared and analyzed the discrete roles of SIRT2 in different conditions, and provided possible explanations for its paradoxical functions. In the future, the rapid growth in SIRT2 research may clarify its impacts on neurological disorders and develop therapeutic strategies targeting this protein.

## Introduction

The mammalian sirtuin family consists of seven members, sirtuins 1 to 7 (SIRT1–SIRT7), which are classified by their highly conserved central nicotinamide adenine dinucleotide (NAD^+^)-binding and catalytic domains. Their diverse functions are based on different enzymatic activities, unique binding partners, substrates, and distinct subcellular localization and expression patterns (Haigis and Sinclair, 2010). Each sirtuin resides in its preferable cellular compartment. SIRT1, SIRT6, and SIRT7 are chiefly found in the nucleus, whereas SIRT3, SIRT4, and SIRT5 are primarily found in the mitochondria (Haigis and Sinclair, 2010). SIRT2 is dominantly localized in the cytoplasm and is able to enter the nucleus to interact with histone H4 Lys 16 (H4K16) during mitosis (Vaquero et al., 2006), suggesting its essential role in chromatin condensation. α-Tubulin, a key component of the skeleton of microtubes, is also an important substrate of SIRT2. Acetylation of α-tubulin by SIRT2 is closely related to brain aging and neurological disorders (Li et al., 2007). SIRT2 is abundantly expressed in the brain by both neurons and oligodendrocytes, confined to the cytoplasm of these postmitotic cells (Harting and Knöll, 2010). Therefore, it is reasonable to assume that SIRT2 plays a vital role in neurological disorders.

Previous studies have corroborated that specific SIRT2 single-nucleotide polymorphisms (SNPs) correlate to the risk of a number of neurodegenerative diseases, like Alzheimer’s disease (AD) (Polito et al., 2013; Porcelli et al., 2013; Xia et al., 2014; Cacabelos et al., 2019; Shen et al., 2020) and Parkinson’s disease (PD) (Wang et al., 2018; Chen et al., 2019) under certain conditions. Recent studies demonstrated that SIRT2 participates in even more pathological processes such as frontotemporal dementia (FTD) (Spires-Jones et al., 2012), stroke (Shu et al., 2019), and brain injury (Wang et al., 2016). Although these diseases have different etiologies, they share some common pathological characteristics: neuroinflammation, synaptic dysfunction, metabolism abnormality, oxidative stress, etc., all of which can be affected by SIRT2. Some characteristics are only observed in specific diseases, such as amyloid beta (Aβ) aggregation and tau protein phosphorylation.

This review not only emphasized and compared the shared characteristics impacted by SIRT2 but also discussed unique SIRT2 functions in each diseased condition. First, we introduced the biochemistry and physiology of SIRT2, then described the mechanisms SIRT2 plays in different neurological disorders, analyzed why SIRT2 has paradoxical impacts on similar pathological processes, and discussed the possibility of developing drugs targeting SIRT2.

## Biochemistry of SIRT2

Sirtuins are lysine deacetylases (KDACs) and previously classified as class III histone deacetylases (HDACs). Unlike classes I, II, and IV that are dependent on Zn^2+^, they take NAD^+^ as a cofactor and herein manifest catalytic activities rather than deacetylation (Schiedel et al., 2018). Sirtuins are also known to catalyze ADP-ribosylation (Frye, 1999), which is especially prominent for SIRT2.

The chemical structure of SIRT2 is fully revealed in 2001, when Finnin et al. (2001) discovered that SIRT2 has a 304-amino acid catalytic core and a 19-residue N-terminal helical extension. The SIRT2 catalytic core has two domains: the larger one is a variant of the Rossmann fold, and the smaller one consists of the zinc-binding and the helical modules (Finnin et al., 2001). At the interface of the two domains, there is a large grove, which includes the NAD binding site and presents the HDAC activity.

In general, there are three explanations for the deacetylation mechanism, among which the ADPR peptidyl-imidate one is well accepted with substantial and solid evidence to support it. This mechanism shows that the process of deacetylation begins with the release of nicotinamide from NAD^+^ and the formation of a C1′-*O*-alkylamidate intermediate from the nucleophilic addition of the acetyl oxygen to the 1′-carbon of the nicotinamide ribose. The *N*-ribose 2′-hydroxyl group activated by a conserved catalytic His residue then attacks the previous intermediate carbon to produce a 1′2′-cyclic intermediate, which will eventually be resolved by a base-activated water molecule to generate the 2′-*O*-acetyl-ADP-ribose and deacetylated lysine (Sauve, 2010; Moniot et al., 2013). The characteristics of the structure and biological reaction of SIRT2 lay foundations for the design and synthesis of SIRT2 modulators.

## Physiology of SIRT2

### SIRT2 in Cell Cycle, Cell Proliferation, and Cell Migration

SIRT2 is mainly present in the cytoplasm but enriched in the nucleus during the cell cycle (North and Verdin, 2007a), implicating its close relationship with cell cycle and cell proliferation. SIRT2 and its yeast counterpart HDAC Hst2 (Hst2) have preferences for H4K16Ac, which peaks at the S phase and dramatically drops in the G2 phase during the cell cycle. Loss of SIRT2 produces a high level of H4K16Ac during mitosis, a delay in S-phase/M transition and abnormal levels of H4K16Ac in heterochromatic foci (Vaquero et al., 2006). The deacetylation of H4K16 by SIRT2 leads to a series of downstream reactions. For example, SIRT2 suppresses the expression of NEDD4 E3 ubiquitin protein ligase (NEDD4), and the latter targets Myc oncoproteins for ubiquitination and degradation. Consequently, the inhibitory effect of NEDD4 on oncogenesis is reduced (Liu et al., 2013). In another study, SIRT2 regulates monomethylation of H4K20 (H4K20mel) and determines the levels of H4K20me2/H4K20me3 throughout the cell cycle. SIRT2-deficient animals exhibit genomic instability and chromosomal aberrations and are prone to tumorigenesis (Serrano et al., 2013), reflecting opposite roles of SIRT2 in cell proliferation and tumorigenesis. This paradox has been reported in other studies as well: the pharmacological blockade of SIRT2 activity or downregulation of SIRT2 expression with small interfering RNA (siRNA) counteracted the inhibitory effect of resveratrol on cell proliferation (Sayd et al., 2014), while another study demonstrated that the SIRT2 inhibitor AGK2 suppressed cell growth and resulted in G1-phase arrest by inhibiting the expression of cyclin-dependent kinase 6 (CDK6) and/or cyclin-dependent kinase 4 (CDK4) (Kim et al., 2016). This suggests that SIRT2 is involved in a complex network regulating cell cycle and exerts discrete functions under different conditions.

SIRT2 correlates to cell cycle checkpoints as well. It has been proved that SIRT2 curbs microtubule poison-induced hyperploid cell formation by blocking the entry to chromosome condensation (Inoue et al., 2007). SIRT2 deficiency contributes to fragility to replication stress, spontaneous accumulation of replication protein A to foci and the chromatin, and a G2/M checkpoint deficit by regulating the activity of cyclin-dependent kinase 9 (CDK9) (Zhang et al., 2013). Another study corroborated that the mutation of serine 368 in SIRT2 reduces hyperploidy in cells under mitotic stress in the presence of microtubule poisons, while SIRT2 overexpression mediates a delay in cellular proliferation by enhancing the level of serine 368 phosphorylation (North and Verdin, 2007b). SIRT2 also affects mitosis by triggering Golgi fragmentation and impairing Golgi assembly (Zhang et al., 2019). SIRT2 deficiency or overexpression results in impairments in nuclear envelope reassembly and acetylation as well as phosphorylation of the ankyrin repeat and LEM domain containing 2 (ANKLE2) throughout the cell cycle (Kaufmann et al., 2016). Apart from cell proliferation and cell cycle, SIRT2 also induces cell detachment and halts migration of mouse embryonic fibroblasts (MEFs) possibly by changing the stability of microtubules due to differential acetylation (Pandithage et al., 2008). The same conclusion is also drawn from another study which showed that SIRT2 hampers cell motility by curbing actin polymerization through heat shock protein 90 (HSP90) destabilization and subsequent repression of the LIM domain kinase 1 (LIMK1)/cofilin pathway (Min et al., 2018). However, Kim et al. (2016) proved that SIRT2 inhibition by AGK2 suppresses migration by compromising the stability of the heat shock transcription factor 1 (HSF1) protein. Dispute on SIRT2 in cell migration has encouraged further research.

### SIRT2 in Calorie Restriction, Metabolism, and Aging

The relationship between calorie restriction (CR) and aging dates back to 1986 when Weindruch et al. (1986) discovered the antiaging effects of dietary restriction. Some people attribute this phenomenon to low reactive oxygen species (ROS), and others assume that the radical shift in the metabolic strategy in cells may account for it (Guarente, 2000). To elucidate the detailed mechanism, Guarente (2000) proposed that Sir2 proteins may link the metabolic rate to the pace of aging by sensing NAD levels and generating the mandated level of chromatin silencing. This assumption has been corroborated later. Only yeast that expresses Sir2 and nicotinate phosphoribosyltransferase (NPT1) show life span extension under the CR condition (Lin et al., 2000), suggesting that Sir2p and NAD are necessary for life span extension induced by CR. The authors assumed that this may be linked to the shunting of carbon metabolism toward the mitochondrial tricarboxylic acid cycle and the concomitant increase in respiration (Lin et al., 2002). Another study verified the above findings and demonstrated that the overexpression of Sir2 and deletion of the fork barrier binding protein (Fob1) contribute to increased longevity induced by CR. The authors interpreted this as an outcome of differences in approaches to achieve CR and yeast strain background (Kaeberlein et al., 2004). Though Sir2 synergistically increases CR-induced replicative life span extension, a study found that Sir2 exerts the opposite effect on the chronological life span of yeast. In this study, lack of Sir2 along with CR showed a dramatic chronological life span extension (Fabrizio et al., 2005). The potential mechanism underlying this inconsistency was discussed in another study which stated that the regulatory function of Sir2 in chronological aging responds to nutrient cues including glucose, possibly through Sir2-dependent modification of chromatin or deacetylation of a non-histone protein (McCleary and Rine, 2017).

In addition to studies on yeast, more experiments were conducted on species like *Drosophila* and other eukaryotes. *Drosophila* with low-calorie intake exhibits high spontaneous activities, which are dependent on the presence of dSir2 (Parashar and Rogina, 2009). Another study concluded that Sir2 in the body is a key regulator of organismal energy homeostasis required for maintaining the metabolic regulatory network across tissues (Banerjee et al., 2013). In a mouse model, SIRT2 regulates insulin-mediated glucose uptake by deacetylating the TUG (encoded by the Aspscr1 gene) peptide (Belman et al., 2015).

Though it is widely accepted that Sir2 and its homolog present synergistic functions in CR-mediated longevity, some researchers showed results that CR-mediated longevity is not dependent on Sir2 (Tsuchiya et al., 2006; Mei and Brenner, 2015), which needs further validation.

## Pathological Participation

### Neuroinflammation and Oxidative Stress

Neuroinflammation is ubiquitously present in neurological disorders. It directly impacts neuronal apoptosis, which results in acute neural damage or neuron death and accelerates long-term neurodegeneration (Lyman et al., 2014). Glial cells, including astrocytes and microglia, along with chemokines and cytokines, play a vital role in neuroinflammation (Perry et al., 2010; Ransohoff, 2016). Neuroinflammation is closely associated with oxidative stress (Akbar et al., 2016). The latter can be triggered by protein aggregates, impair mitochondrial function, and injure neurons, leading to DNA damage as well as structural alteration of proteins and lipids (Zhang et al., 2020).

The relationship between SIRT2 and neuroinflammation has been inconclusive for a long time. SIRT2 inhibition by a high concentration of AGK2 was reported to block Aβ-triggered inflammation *in vitro* (Scuderi et al., 2014). SIRT2-knockout mice manifest less inflammatory response triggered by lipopolysaccharides (LPS) (Lee et al., 2014), mainly due to inhibition of nuclear factor kappa-B (NF-κB) activation by deficiency of SIRT2. Consequently, the phosphorylation and degradation of IκBα was decreased, resulting from decreased p65 phosphorylation and nuclear translocation (Lee et al., 2014). Application of AGK2 inhibits inflammatory signals triggered by LPS as well (Wang et al., 2016; Harrison et al., 2018), which is possibly attributed to decreased nuclear translocation of NF-κB (Wang et al., 2016). Interestingly, another study showed similar results in BV2 microglia cells (Chen et al., 2015), but altered nuclear translocation of NF-κB was not observed. This discrepancy may be the result of different experimental conditions or tissue specificity, with one study selecting bone-derived macrophage (Lee et al., 2014) and another selecting BV2 microglia (Chen et al., 2015). Apart from its direct regulating role, SIRT2 is also able to curtail the anti-inflammatory effect of Treg cells by modulating the expression of immunosuppression-associated molecules including forkhead box P3 (Foxp3), indirectly ameliorating neuroinflammation in the middle cerebral artery occlusion (MCAO) mouse model (Shu et al., 2019). Though the majority of studies support the anti-neuroinflammation role of SIRT2, conflicting results were also reported. In the controlled cortical impact (CCI) injury model mimicking brain injury, inhibition of SIRT2 by AK-7 exacerbated inflammatory response, which may result from elevated NF-κB activation and increased acetylation and activation of the p65 subunit (Yuan et al., 2016). The following factors may explain the conflicting results: (1) difference in the animal model used (mainly LPS-induced inflammation models or the CCI injury model); (2) the endurance of the trigger applied to the animal model; (3) approaches to silence SIRT2; and (4) tissue specificity.

Studies have shown that targets of SIRT2 deacetylation have important roles in antioxidant- and redox-mediated cellular homeostasis both *in vitro* and *in vivo* (Gomes et al., 2015). In SH-SY5Y cells, elevated SIRT2 protected cells from rotenone- or diquat-induced cell death through increasing the expression of antioxidant substances such as superoxide dismutase 2 (SOD2) (Singh et al., 2017). *In vivo*, 13-month-old mice lacking SIRT2 exhibit energy failure due to mitochondrial depletion, and redox dyshomeostasis (Fourcade et al., 2017). Overall, SIRT2 acts as a protectant for cells to combat against oxidative stress (Singh et al., 2018). However, another study claimed that inhibiting SIRT2 by AK-7 may protect 1-methyl-4-phenyl-1,2,3,6-tetrahydropyridine (MPTP) mice from redox dysfunction (Guan et al., 2016). Therefore, more comprehensive studies are required to clarify the exact role of SIRT2 in oxidative stress.

### Synaptic Changes and Axonal Degeneration

Synaptic changes and axonal dysfunction directly contribute to cognitive impairment and behavior changes. SIRT2 is one of the key factors modulating hippocampus function such as cell proliferation, memory formation, and neuroblast differentiation (Yoo et al., 2015; Jung et al., 2016). Nicotinamide is confirmed to be protective in the slow Wallerian degeneration (Wlds) mouse model, and its function does not relate to SIRT1 (Wang et al., 2005). Though participation of SIRT2 was not tested, it was expected that inhibition of SIRT2 is related to axonal degeneration. This hypothesis was verified by showing that SIRT2 overexpression abrogates microtubule hyperacetylation and resistance to axonal degeneration, while SIRT2 silencing by siRNA enhances microtubule acetylation and resistance to axonal degeneration in WldS mice (Suzuki and Koike, 2007). Though it is known that inhibition of SIRT2 protects axons, middle-aged mice lacking SIRT2 exhibit axonal degeneration and locomotor dysfunction (Fourcade et al., 2017). The opposite roles of SIRT2 may arise from the difference in the choice of mouse models or the approaches to induce axonal dysfunction. Sirtuin-knockout mice exhibit axonal dysfunction (Fourcade et al., 2017) or less voluntary differentiation of dopaminergic neurons due to knockout of the sirtuin gene (Szego et al., 2017), whereas the axonal degeneration in WldS mice is due to the transection of axons (Suzuki and Koike, 2007). We assume that SIRT2 may be necessary for normal axonal formation but functions differently after that.

As for SIRT2 on synaptic plasticity, SIRT2 functions as an AMPA receptor (AMPAR) deacetylase (Wang et al., 2017). It interacts with GluA1, a subunit of AMPAR. The downregulation or absence of SIRT2 increases GluA1 acetylation by competitively decreasing GluA1 ubiquitination, through which AMPAR trafficking and proteostasis are regulated and learning and memory capabilities are influenced. There are few studies focusing on SIRT2 and synaptic plasticity, and more investigations are in demand.

### Autophagy, Apoptosis, and Programmed Necrosis

SIRT2 influences autophagy and apoptosis mainly by regulating microtubule-related proteins and alleviating the toxicity of misfolded proteins. Pharmacological inhibition of SIRT2 prevents microtubules from impairment by selectively enhancing α-tubulin acetylation in sPD cells, and SIRT2-knockout mice also maintain stable microtubule assembly after being exposed to MPP +. The stability of microtubules is essential for the clearance of misfolded proteins and normal autophagic flux (Esteves et al., 2018). α-syn, AβPP, and tau protein can lead to mitophagy and autophagy, and SIRT2 inhibition attenuates the toxicity of these misfolded proteins, indirectly participating in the regulation of autophagy. Deletion of Sir2 in the yeast abolishes autophagy and mitophagy and consequently rescues cells (Sampaio-Marques et al., 2012). In microglial BV2 cells, SIRT2-induced autophagy was also found as a result of AGK2-induced ATP decline (Li et al., 2013). A similar phenomenon was observed in mammalian models (de Oliveira et al., 2017). In addition, SIRT2 has been shown to enhance apoptosis in the MPTP model of PD through deacetylating Foxo3a and increasing levels of Bim RNA and protein, exacerbating MPTP-induced nigrostriatal damage (Liu et al., 2014). Inhibition of SIRT2 either by AK-1 or by SIRT2 knockout restored microtubule stability and improved autophagy, favoring cell survival through eliminating toxic Aβ oligomers (Silva et al., 2017). In addition to clearing Aβ, SIRT2 is able to impact the phosphorylation of tau and autophagic influx in AD (Esteves et al., 2019). The neuroprotective effects of SIRT2 inhibition have also been found in ischemic stroke, which is mediated by the downregulation of AKT/FOXO3a and mitogen-activated protein kinase (MAPK) pathways (She et al., 2018).

Accumulating evidence has shown that necrosis is also a programmed process (Vandenabeele et al., 2010). SIRT2 regulates this process in ischemic stroke. Deletion or knockdown of SIRT2 blocks cellular necrosis induced by tumor necrosis factor α (TNF-α) through preventing the formation of the receptor-interacting protein 1 (RIP1)–receptor-interacting protein 3 (RIP3) complex (Narayan et al., 2012). There are relatively few studies available in this field, and future research is required to confirm the role of SIRT2 in necrosis.

## SIRT2 and Neurological Disorders

### Alzheimer’s Disease

The concomitant presence of Aβ and tau is still the classical characteristics of AD (Scheltens et al., 2016). SIRT2 influences the amyloid beta-protein precursor (APP) transformation process and tau protein phosphorylation. Inhibition of SIRT2 by AGK2 in H4-SW cells reduced levels of Aβ40 and Aβ42 as well as soluble AβPPβ (sAβPPβ), whereas inhibition of SIRT2 by AK-7 in both APP23 and 3xTg transgenic mice increased the level of soluble AβPPα (sAβPPα), indicating that SIRT2 may regulate the activity or quantity of α and β secretases (Biella et al., 2016). Intriguingly, *in vivo* experiment did not find significant changes in Aβ40 and Aβ42 after inhibiting SIRT2, which might be attributed to environmental differences between *in vivo* and *in vitro* conditions (Biella et al., 2016). Apart from the direct impacts of SIRT2 inhibition on Aβ, SIRT2 loss of function indirectly promoted the clearance of Aβ by restoring autophagy (Silva et al., 2017). Though inhibition of SIRT2 improved memory in murine models (Biella et al., 2016), clinical evidence did not show correlations between the level of SIRT2 mRNA and cognitive performance of patients, and no difference in the level of SIRT2 mRNA in the periphery between normal senior people and AD patients was found (Wongchitrat et al., 2019). This inconsistency between animal experiments and clinical trials implies the complicated relationship between AD and SIRT2.

When investigating the role of SIRT2 in AD pathogenesis, ideal animal models ensure the significance of research findings and the possibility of clinical translation. One of the animal models that researchers usually use is the 3xTg mouse model. Though this mouse model is not perfect for examining the relationship between SIRT2 and cognitive impairment because of the presence of Aβ deposit in the 3xTg model, AD models with sole tau pathology are hard to find. Transgenic tau models like rTg4510 and PS19 are not applicable to AD research for no tau mutations relate to AD. Though humanized tau (htau) models almost perfectly recapitulate the characteristics of tau pathology of AD, their behavior deficits and pathological phenotypes are very mild, which limits their application (Jankowsky and Zheng, 2017).

Inhibition of SIRT2 by AK-7 increased the steady-state level of tau protein, which assisted in stabilizing microtubules in a healthy neuron, which may account for alleviated neurodegeneration in AD models. But this research failed to measure the level of phosphorylated tau due to the relatively young age of mice (Biella et al., 2016). Another study found that nicotinamide, a competitive inhibitor of the sirtuins, can restore cognitive function by selectively reducing a specific phospho-species of tau (Thr231) (Green et al., 2008); SIRT2 inhibition by AK-1 is corroborated to decrease phospho-tau as well (Esteves et al., 2019). Interestingly, it has been demonstrated in this study that the effect of nicotinamide on cognition is not related to changes of Aβ (Green et al., 2008). Discrepancy in findings by Biella et al. (2016) and Green et al. (2008) may be partially due to the selection of different inhibitors of SIRT2. It is known that NAD is not a specific SIRT2 inhibitor and that the involvement of other sirtuin family members cannot be excluded. In the study of Green et al. (2008), quantities of APP and its metabolites were measured, but the distribution of Aβ deposit was not tested, and this may directly contribute to cognitive impairment or indirectly participate in tau pathology and cognitive impairment.

SIRT2 not only influences AD by intervening with the process of Aβ aggregation and tau phosphorylation through regulating the Aβ/tau-mediated autophagy but also impacts AD by increasing the clearance of misfolded proteins. Inhibition of SIRT2 either by AK-1 or by SIRT2 knockout restored microtubule stabilization and improved autophagy, favoring cell survival through eliminating toxic Aβ oligomers (Silva et al., 2017). In addition to Aβ, SIRT2 was shown to impact the phosphorylation of tau and autophagic influx in AD (Esteves et al., 2019).

Given that AD may benefit from SIRT2 inhibition, scientists have been testing if a selective SIRT2 inhibitor may be a promising drug target for AD. Diaz-Perdigon et al. (2020) found that early SIRT2 inhibition by a selective SIRT2 inhibitor 33i might be beneficial for preventing age-related cognitive deficits, neuroinflammation, and AD progression in the senescence-accelerated mouse prone-8 (SAMP8) model, which provided new insights into AD therapy.

### Parkinson’s Disease

PD is characterized by progressive loss of dopaminergic neurons and accumulating Lewy bodies (LBs) in the substantia nigra (SN). The pathologically misfolded protein α-syn is the main component of LBs (Ghosh et al., 2017). α-Syn is acetylated on lysines 6 and 10, and these residues can be deacetylated by SIRT2, implying the potential role of SIRT2 in α-syn acetylation, aggregation, and autophagy (de Oliveira et al., 2017). SIRT2 inhibition increased the level of acetylated α-syn, decreased the aggregation and toxicity of α-syn, and elevated the activity of alkaline phosphatase (ALP), which led to clearance of α-syn aggregates (de Oliveira et al., 2017). Pharmacological inhibition of SIRT2 by AGK2 or AK-1 rescued α-syn-mediated toxicity by transforming toxic, submicroscopic a-syn oligomers into larger inclusions, but why SIRT2 inhibition affected α-syn aggregation is not clear. Though researchers assume that an increase in acetylated α-tubulin by SIRT2 may account for it, they have not validated this hypothesis (Outeiro et al., 2007). A later study found that the absence of Sir2, a homologous-to-mammalian sirtuin protein, can alleviate α-syn toxicity by inhibiting mitophagy/autophagy (Sampaio-Marques et al., 2012). In this study, it was shown that α-syn can induce detrimental autophagy/mitophagy and superoxide anion accumulation in the presence of ATG11/ATG32 (two scaffold proteins closely related to autophagy/mitophagy) (Sampaio-Marques et al., 2012). Therefore, it is reasonable to speculate that absence of Sir2 may be related to oxidative stress, and this was confirmed by Guan et al. (2016) who showed that SIRT2 inhibition by AK-7 improved MPTP-induced neurochemical and behavioral deficits by ameliorating dysfunction of the redox network. In contrast, another study demonstrated that SIRT2 inhibition exacerbated α-syn aggregation under the condition of oxidative stress caused by diquat/rotenone (Singh et al., 2017). Though the majority of studies claimed the protective role of SIRT2 inhibition in α-synucleinopathy, the detailed mechanism is still unclear, which demands more investigations.

SIRT2 reduces the toxicity of misfolded proteins by accelerating the process of clearance and regulating the process of autophagy. Pharmacological inhibition of SIRT2 prevented microtubules from being impaired by selectively enhancing α-tubulin acetylation in sPD cells, and SIRT2-knockout mice also maintained stable microtubule assembly after being exposed to MPP +. The stability of microtubules is essential for the clearance of misfolded proteins and normal autophagic flux (Esteves et al., 2018). Though normal autophagic flux is protective, excessive autophagy is detrimental. Deletion of Sir2 in the yeast abolishes autophagy and mitophagy and consequently rescues cells (Sampaio-Marques et al., 2012), and a similar phenomenon was observed in mammalian models (de Oliveira et al., 2017). However, SIRT2 is not always protective. A study reported that SIRT2 aggravated MPTP-induced nigrostriatal damage by enhancing apoptosis in the MPTP model (Liu et al., 2014). Therefore, it is necessary to investigate the exact role of SIRT2 in autophagy of PD.

### Brain Injury

Brain injury consists of penetrating and non-penetrating injuries. The main pathophysiological changes include autoregulatory disturbances, abnormal glutaminergic and GABAergic functions, massive accumulation of K^+^ in the extracellular space, the influx of Na^+^ and Ca^2+^ through glutamate receptor-gated ion channels, and uniform decline in oxygen and glucose metabolism (McGinn and Povlishock, 2016). Given that SIRT2 is closely related to inflammation and autophagy, both of which are present in brain injury, SIRT2 may be involved in the process of brain injury. A study has discovered that inhibition of SIRT2 by AK-7 led to exacerbation of brain edema and neuroinflammation in an experimental traumatic brain injury (TBI) model CCI (Yuan et al., 2016), whereas another study has reported conflicting results. It was found that administration of the SIRT2 inhibitor AGK2 significantly decreased LPS-induced increase in CD11b, TNF-α, and IL-6 and blocked LPS-induced nuclear translocation of NF-κB and signaling pathways related to activation of autophagy (Wang et al., 2016).

### Stroke

Infarction of the central nervous system refers to cell death of the brain, cell death of the spinal cord, or cell death due to ischemia, and diagnosis is based on imaging, clinical, and other objective evidence (Sacco et al., 2013). It is classified into ischemic and hemorrhagic stroke, and ischemic stroke is more common. In stroke, SIRT2 presents contradictory functions as well. SIRT2 impairs the anti-inflammatory effect of Treg cells and indirectly impacts the process of stroke in the MCAO model (Shu et al., 2019). Inhibition of SIRT2 by AK-7 rescued neurological function and decreased stroke volume via p38 activation. SIRT2 has also been verified to regulate programmed necrosis in ischemic stroke. Deletion or knockdown of SIRT2 blocked cellular necrosis induced by TNF-α through preventing the formation of the RIP1–RIP3 complex (Narayan et al., 2012). However, the neuroprotective effect of SIRT2 inhibition on ischemic stroke has also been reported, which is mediated by downregulation of AKT/FOXO3a and MAPK pathways (She et al., 2018).

### Other Neurological Diseases

FTD is classified as a neurodegenerative disease. It mainly manifests by progressive decline in behavior and language caused by focal degeneration of frontal and anterior temporal lobes (Shinagawa, 2013). FTD is also defined as a tau-associated disease. Given that SIRT2 can influence the process of tau pathology, it is reasonable to deduce that SIRT2 may also play an important role in FTD. SIRT2 inhibition by AK-1 is reported to be non-toxic and to prevent neurodegeneration in the rTg4510 brain by decreasing neuronal loss (Spires-Jones et al., 2012). There are few studies focusing on SIRT2 and FTD, and further investigations are in demand.

## Crosstalk Between SIRT2 and Phenotypes

**FIGURE 1:**
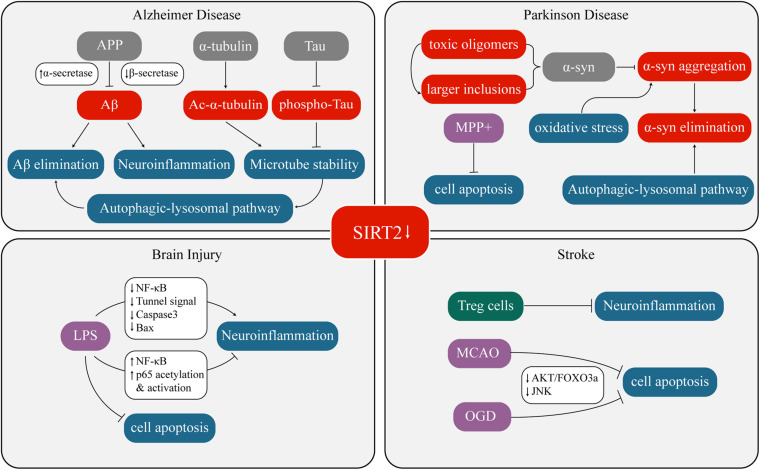
Crosstalk between SIRT2 and neurological diseases.

Overexpression or inhibition/knockout/silencing of SIRT2 can lead to changes in different phenotypes. These phenotypes interact with each other under certain circumstances as well. Figure 1 and Table 1 have concluded the sophisticated relationship between SIRT2 and various phenotypes.

**TABLE 1:** w

Pathology	Background	Sirt2 downregulation	Pathway	Downstream changes	References
Aβ	H4-SW cellsAPP23 mice3xTG-AD micesAD cellsC57-BL6 mice	AGK2AK-7AK-7 AK-1SIRT2 KO/AK-1	NA NA NA NA NA	↓Aβ40 and Aβ42 ↑α secretase, ↓β secretase, ↓Aβ production ↓microtubule acetylation, ↓ALP impairment ↑microtubule stabilization, ↑autophagy, ↑cell survival, ↓toxic Aβ oligomers	Biella et al., 2016; Silva et al., 2017
Tau	3xTg-AD mice P301 cells 3xTg-AD mice rTg4510 mice	AK-7 AK-1 Nicotinamide AK-1	NA NA ↑p25 NA	↑steady-state level of tau, ↑microtubule stabilization ↑Ac-α-tubulin ↓Phospho-tau ↓Thr231-phosphotau, ↓monoubiquitinated tau, ↑tau degradation No effects on NFTs formation or stability	Biella et al., 2016;Esteves et al., 2019;Green et al., 2008;Spires-Jones et al., 2012
α-synuclein	Human neuroglioma cells (H4) Drosophila PD modelH4 cellsMPTP model SH-SY5Y cells	SiRNA AGK-2 AK-1AGK-7 AK-1/AGK-2 shRNASIRT2 KOAGK-2	NA NA NA NA NA NA NA FOXO3a pathway	↓α-syn toxicity↓α-syn toxicity, ↑ larger inclusions formation↓α-syn toxicity, ↑ larger inclusions formation (less potent) No effects dose-dependent rescue of dorsomedial neurons↓SynT-positive inclusions, ↑ acetylated aSyn, ↑ALP (autophagy lysosome pathway) activity, ↑clearance of α-syn aggregates ↑TH positive neurons ↑ rotenone/diquat induced oxidative stress, ↑oxidative stress induced α-syn aggregation	Outeiro et al., 2007;de Oliveira et al., 2017; Singh et al., 2017
Neuro- inflammation	BMDM cells RAW cells Primary rat astrocytes microglial BV2 cellsC57BL/6 mice (brain injury model) C57BL/6 mice (CCI injury model)N27 cells Treg cells from ipsilateral hemispheres of MCAO model senescence-accelerated mouse prone-8 (SAMP8) model	SIRT2 KO siRNA/control RNA/AGK-2/AK-1 AGK-2 SIRT2 siRNA AGK-2 AK-7 AGK-2 AGK-2 33i	NFκB pathwayNA NA NFκB pathwayNFκB pathway NA NANA	↓LPS-induced inflammatory reactions↓Aβ-induced inflammation ↓LPS-induced inflammatory reactions ↓LPS-induced inflammatory reactions, ↓LPS-induced apoptosis ↑AQP-4 expression, cerebral edema and BBB disruption, ↑CCI injury model induced inflammation ↑histone acetylation, ↓LPS-induced inflammatory reactions ↑immunoregulatory activity of infiltrating Treg cells ↑glutamate receptor subunits GluN2A, GluN2B, and GluA1, ↓inflammation-related factors expression	[Bibr B33][Bibr B62][Bibr B8][Bibr B77][Bibr B85][Bibr B25][Bibr B67] Diaz-Perdigon et al., 2020
Oxidative stress	MPTP model SH-SY5Y cells C57BL/6J cells	AK-7 AGK-2 SIRT2 KO	NA FOXO3a pathway NA	↑GSH, ↓MDA, ↓oxidative stress ↑ rotenone/diquat induced oxidative stress↓endogenous antioxidant defense (Sod1, Sod2, Gpx1, and Cat), ↑oxidative stress	[Bibr B22][Bibr B70][Bibr B17]
Synaptic changes	DIV11 cultured hippocampal neurons cultured cortical neurons Sprague-Dawley rat	B2 siRNAB2 SIRT2 KO	NANA NA NA	↑AMPARs expression↓internalization, ↑ AMPARs mediated synaptic transmission ↑ AMPARs acetylation, ↓AMPARs ubiquitination ↓LTP and LTD, ↓synaptic plasticity, ↓learning and memory	Wang et al., 2017
Axonal degeneration	Wallerian degeneration slow (Wlds) mice cerebellar granule cells from Wlds mice C57BL/6J mice	Nicotinamide Nicotinamide/SIRT2 siRNA SIRT2 KO	NA NA NA	↓axonal degeneration, ↑microtubule acetylation, ↑resistance to axonal degeneration ↓tubulin deacetylation, ↑resistance to axonal degeneration but not cell body ↑axonal degeneration	[Bibr B79][Bibr B72][Bibr B17]
Autophagy & Apoptosis	BV2 cells C57BL/6 mice SH-SY5Y cells C57BL/6 mice (brain injury model) H4 cells sAD cells C57-BL6 mice primary cortical neurons underlying OGD C57BL/6NTac mice with MCAO surgery sPD cells	AGK-2 SIRT2 KO SIRT2 shRNA AGK-2 shRNA AK-1 SIRT2 KO/AK1 AK-1 AGK-2 AGK-2 AK-1	PARP activation NA Foxo3a pathway NFκB pathway NA NA NA FOXO3a/MAPK pathway FOXO3a/MAPK pathway NA	↓ATP, ↑cell death ↓apoptosis ↓MPP-induced apoptosis ↓LPS-induced apoptosis ↑ALP activity ↓ALP impairment ↑autophagy, ↑cell survival, ↓toxic Aβ oligomers ↓apoptotic cell death ↓infarct size, ↑neurological outcome, ↓apoptotic factors ↑Ac-α-tubulin, ↑stability of microtubes, ↑normal autophagic flux	[Bibr B35][Bibr B38][Bibr B77] [Bibr B10][Bibr B68] [Bibr B64][Bibr B13]

## Modulation of SIRT2 as a Drug Target and Clinical Implications

As stated above, SIRT2 might play a negative role in neurological diseases, and it is a hot spot to search for disease-modifying SIRT2 modulators. This section mainly focused on SIRT2 modulators targeting neurological diseases.

The formation of a C1’-*O*-alkylamidate intermediate is important in the deacetylation process of SIRT2. Nicotinamide is a potent inhibitor of this reaction because it can bind to and attack the intermediate (Alcaín and Villalba, 2009). Green et al. (2008) verified the effect of nicotinamide in the 3xTg AD mouse model: nicotinamide decreased the level of phosphorylated tau protein and increased its degradation. Nicotinamide is also protective in axonal degeneration by elevating the acetylation of microtubules (Wang et al., 2005) or reducing the deacetylation of tubulin (Suzuki and Koike, 2007).

As previously discussed, SIRT2 modulators AGK2, AK-1, and AK-7 have been widely explored in different neurological models (Table 1). AK-1 has been reported to stabilize microtubes, improve cell survival, alleviate the toxicity of Aβ oligomers (Silva et al., 2017), and increase the level of acetylated α-tubulin as well as decrease tau phosphorylation (Esteves et al., 2019) in AD. However, it has been proven to be ineffective on formation of neurofibrillary tangles (NFTs) or stability in FTD though it possesses the ability to prevent neurodegeneration in general (Spires-Jones et al., 2012). AGK2 and AK-7 are also effective in improving cognitive performance in AD by regulating the metabolism of AβPP (Biella et al., 2016). In PD, AK-1 and AGK2 were demonstrated to modify the morphology of inclusion bodies, rescue α-syn toxicity, and dose-dependently rescue dorsomedial neurons, whereas AK-7 did not have such protective effects (Outeiro et al., 2007). Conflicting results were also reported. A study showed that AGK2 aggravated oxidative stress and the resulting α-syn aggregation in PD (Singh et al., 2017). Another study demonstrated that AK-7 improved neurological functions by alleviating oxidative stress (Guan et al., 2016). In brain injury and stroke, SIRT2 modulators usually take their effect through regulating neuroinflammation and autophagy, but a definite conclusion on their efficacy has not been reached (Wang et al., 2016; Yuan et al., 2016; She et al., 2018; Shu et al., 2019), mainly due to lack of substantial data.

Besides nicotinamide, AK-1, AK-7, and AGK2, new highly selective SIRT2 inhibitors have emerged. 3-((2-Methoxynaphthalen-1-yl)methyl)-7-((pyridin-3- ylmethyl) amino)-5,6,7,8-tetrahydrobenzo[4,5]thieno[2,3-d]pyrimidin-4(3*H*)-one (ICL-SIRT078) showed a neuroprotective effect on parkinsonian neuronal cell death in the lactacystin-induced N27 cell model (Di Fruscia et al., 2015). Another new class of SIRT2 inhibitors, 5-((3-amidobenzyl)oxy)nicotinamides, is corroborated to protect SH-SY5Y cells from α-synuclein aggregation-induced cytotoxicity (Ai et al., 2016).

When it comes to clinical translation, findings in these studies are contradictory, and most of them focused on the relationship between SIRT2 and phenotypes but neglected what roles the SIRT2 modulators eventually play in cognitive/behavioral improvement and neuroimaging changes. Each disease has a series of phenotypes, and improvement in one or two phenotypes does not extrapolate to state improvement in all phenotypes of this disease. Further studies should focus on *in vivo* experiments and take cognitive and/or behavioral improvement and neuroimaging changes as indicators for evaluating the efficacy of SIRT2 modulators.

## Conclusion

In summary, SIRT2 exerts a detrimental effect on majority of neurological diseases, inhibiting SIRT2 by chemical inhibitors, SIRT2 knockout, or siRNA silencing protects the brain. Though SIRT2 inhibitors are promising in curing neurological diseases, we cannot dismiss the paradoxical role of SIRT2 in neuroinflammation, axonal dysfunction, and autophagy. The continuous growth of SIRT2 research will help clarify functions of SIRT2 and accelerate translation of SIRT2 research findings.

## Author Contributions

XC searched literatures, drafted the manuscript, and drew the figure and tables. All authors contributed to the revision of the manuscript and approved the submitted version.

## Conflict of Interest

The authors declare that the research was conducted in the absence of any commercial or financial relationships that could be construed as a potential conflict of interest.
